# Chemical Intolerance Is Associated With Altered Response Bias, not Greater Sensory Sensitivity

**DOI:** 10.1177/2041669520978424

**Published:** 2020-12-20

**Authors:** Linus Andersson, Petra Sandberg, Elisabeth Åström, Moa Lillqvist, Anna-Sara Claeson

**Affiliations:** Department of Psychology, Umeå University, Umeå, Sweden; Department of Radiation Sciences, Umeå University, Umeå, Sweden; Department of Psychology, Umeå University, Umeå, Sweden

**Keywords:** chemical intolerance, chemosensory, idiopathic environmental intolerance, multiple chemical sensitivity, signal detection theory, smell

## Abstract

Chemical intolerance is a surprisingly prevalent condition or affliction characterized by adverse reactions to low levels of chemical, often odorous stimulation. Sufferers often assume that their plight is due to an uncommon sensory acuteness, yet studies repeatedly fail to reveal altered detection thresholds. Here, we investigated whether self-reported chemical intolerance is associated with altered sensory sensitivity or response bias. The sensory acuity (sensitivity; A) and sensory decision rule (criterion; B) to *n*-butanol was assessed using the method of constant stimuli in 82 participants with different degrees of chemical intolerance (low to high). Higher self-reported chemical intolerance was associated with a lower criterion, but not with sensitivity.

Chemical intolerance is a label used to describe adverse reactions to ambient, often odorous chemicals below levels regarded as hazardous (Dantoft, Andersson, et al., 2015). Individuals suffering from this affliction, disease, state, or trait—the characteristics of the phenomenon is debated—thus report that everyday chemical exposure make them sick. It could be the scent of the moisturizing cream used by a friend that triggers reactions, or the lingering traces of cleaning agents used in the public library toilets, or the fabric softener used by a fellow bus passenger (Claeson et al., 2018). Symptoms are diverse (Andersson, Andersson, et al., 2009) and nonspecific but often involves the upper respiratory system (e.g., coughing), mucosae (e.g., nose irritation), and neurocognitive reactions (e.g., headache).

Chemical intolerance is an umbrella term that includes more specific criteria definitions such as multiple chemical sensitivity (Lacour et al., 2005; “Multiple Chemical Sensitivity,” 1999). There is however no generally agreed-upon medical diagnosis of chemical intolerance, or for that matter its subcategories. It is therefore difficult to estimate prevalence, yet several studies suggest that it is surprisingly high—from a few percent to almost one third of the population depending on definition (Karvala et al., 2018). For a more thorough overview of the condition, see Dantoft, Andersson, et al. (2015). Chemical intolerance is often studied dichotomously, that is, as a condition that a person either does or does not have. However, it can also be conceptualized as a continuum where the health impact varies from mild to severe (Haanes et al., 2020).

Sufferers of chemical intolerance often assume that their symptoms are the unfortunate consequence of having a particularly acute sense of smell—that they are the bloodhounds of their families, able to detect even the slightest traces of airborne chemicals. Yet psychophysical studies have repeatedly shown that this assumption is incorrect. Individuals with chemical intolerance do not generally have better noses than others in this regard (Caccappolo et al., 2000; Doty et al., 1988; Kärnekull et al., 2011; Papo et al., 2006). A possible exception is chemicals that are registered by receptors on the trigeminal nerve (cranial nerve V, mediating pungent, burning, chilling, and astringent sensations), where some authors have found deviating function (Andersson, Bende et al., 2009; Claeson & Andersson, 2017), whereas others have not (Caccappolo et al., 2000; Dalton & Hummel, 2000; Papo et al., 2006).

The common procedure used in the published sensory studies on chemical intolerance is to establish absolute detection thresholds for chemical stimuli. This is based on classical threshold theory (Gescheider, 1997), where the threshold is conceptualized as a step-like function producing either signal-present or signal-absent responses. Measuring sensory acuity in this way is often an adequate and efficient method, yet the threshold concept does not capture the inherent ambiguity of sensation. An alternative perspective is provided by signal detection theory (SDT), where the task of the sensory system is described as deciding whether an observation is sampled from an unknown distribution of irrelevant noise, or if a signal of importance is present in this noise (Green & Swets, 1966). Regardless of the signal intensity or the sensitivity of the sensory system, this judgment is always made under uncertainty. The decision depends on a cutoff point, a “criterion,” which is constantly changing based on the costs and benefits of different outcomes (hits, misses, false alarms, and correct rejections). For an overview of psychophysical methods and theories, see, for example, Gescheider (1997) and Wixted (2019).

Sensory sensitivity in SDT terms can be understood as an index of the capacity of receptors, sensory nerves, and relevant brain areas to separate signals from noise. It is assumed to be relatively stable—increasing sensitivity would arguably necessitate a structural alteration in the form of, for example, dendritic sprouting, additional receptors, or larger volume of, for example, the olfactory bulb/sensory projection areas. Although there are studies on the effect of chemosensory training on sensitivity and its physiological substrates (Livermore & Hummel, 2004; Negoias et al., 2017), the results are not always clear. There is as of yet no indications of observable, structural alterations in key chemosensory areas in chemical intolerance, which could be related to the absence of results regarding sensitivity. Changes in the criterion could be a reasonable alternative explanation for why persons with chemical intolerance regard themselves to be good smellers (Andersson, 2012). A low criterion would imply that more signals are detected but also a greater propensity of incorrectly perceiving an odor even when no stimulus is present (i.e., making more false alarms).

The aim of the study is therefore to investigate the association between, on one hand, the degree of self-reported chemical intolerance and, on the other, the ability to separate chemosensory signals from noise (i.e., sensitivity) and the leniency/strictness of the sensory decision rule (i.e., the criterion). Based on the previous empirical evidence and theoretical arguments, we hypothesize that a greater degree of chemical intolerance is associated with a lower criterion, but not higher sensitivity. If the hypotheses hold, they could explain the lack of threshold differences in the literature but also give insight into the common occurrence of chemical intolerance sufferers reporting a particularly good sense of smell.

## Methods

### Participants

Adult participants (43 women, mean age = 38, SD ± 15; 39 men, mean age = 34, SD ± 12) were recruited in two sweeps through advertisement placed at public places such as shopping malls, libraries, and local hospital. The first sweep was for adult individuals in general, while the second was mainly targeted at individuals who regarded themselves as adversely affected by everyday chemical exposure to include a greater variety of chemical intolerance in the sample. Smoking and pregnancy constituted exclusion criteria. The study was conducted in accordance with the Helsinki Declaration and approved by the Umeå Regional Ethics Board (ref.: 2015/99-31Ö and 2016-31-32Ö). Additional information regarding self-reported health status is provided in Table S1.

#### Self-Reported Chemical Intolerance

Degree of self-rated chemical intolerance was assessed using the chemical sensitivity scale (CSS) that is a questionnaire for quantifying self-reported affective/behavioral reactions to everyday odorous/pungent exposure (Nordin et al., 2003). Respondents rate to what degree they agree to 21 statements such as “Even food odors that I normally like will bother me if I am trying to concentrate.” Scores range from 1 to 105, and a high CSS score indicates greater self-reported problems. The CSS has previously been shown to have good reliability (internal consistency: α = .88; test–retest reliability: *r* = .87) and adequate predictive and concurrent validity (Nordin et al., 2003). According to Swedish normative data from Nordin et al. (2004), the CSS has a mean score of 62.3 (SD ± 15.2). The current sample has a slightly lower mean value of 55.4 (SD ± 15.2), a range of 16–98, a Skewness of 0.149, and Kurtosis of –0.267 but was similar to a previous exposure study with comparable sample procedures (Nordin et al., 2017).

### Olfactory Stimuli

The participants’ olfactory sensitivity and criterion were established using a method of constant stimuli procedure (Gescheider, 1997). Stimuli consisted of 60 ml dilutions of the odorant *n*-butanol (99.7% VWR) mixed with water in 500 ml glass flasks, equivalent to dilution steps 6 through 12 described by Cain (1989). The resulting *n*-butanol concentrations were thus in descending order from 6 to 12: 14.4, 6.1, 2.2, 0.67, 0.24, 0.1, and 0.03 mg/m^3^ (Cometto-Muñiz et al., 2003). Blank stimuli consisted of 60 ml pure tap water in three glass flasks. The rationale for using three flasks for blank was to be able to alternate between them to avoid excessing heating by repeated handling, as blanks were presented more often than the different odor stimuli. Flasks were kept in a refrigerator when not in use but were taken out 2 hours prior to testing to reach room temperature. The flasks with *n*-butanol were used for no more than 3 days before exchanging them for new batches, whereas the blank stimuli were changed every day. Nonparametric measures of sensitivity (A) and criterion (B) were calculated according to Zhang and Mueller (2005).

### Procedure

After having expressed their interest in the study, participants were contacted through mail, given additional information and the informed consent form to read through, and were invited to schedule themselves to one of several time slots using an online calendar. They were further briefed about the study once they arrived at the laboratory, were given the opportunity to ask follow-up questions, and signed the informed consent form if they wanted to proceed. The procedure described in this article is one part of a more encompassing data collection.

**Figure 1.**
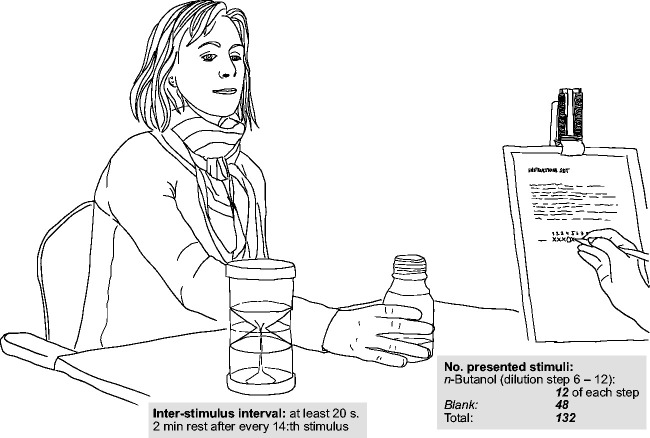
Illustration of the study setup.

The participant and test leader were seated facing each other in a well-ventilated room (see Figure 1). Participants were informed that the task was to decide whether bottles contained either an odorant (*n*-butanol) or just water and were given the opportunity to smell the bottle with the strongest concentration (dilution step 6; 14.4 mg/m3) and a bottle with only water (blank). The test leader then presented the flasks in a randomized order (but which was the same for each participant), one at a time with at least 20-second interstimulus intervals, and with a 2-minute break after each 14th flask. The test leader kept the flasks hidden behind a screen so that the participants could not discern how many flasks were presented, and when they were reused. Each dilution step was presented 12 times, whereas blanks were presented 48 times, for a total of 132 flask presentations. The participant was further encouraged to take one or two sniffs and to not overthink their decision.

### Statistical Analysis

The statistical analyses were carried out using SPSS version 26. Multiple regression was used to examine the main research question, that is, the associations between sensitivity, criterion, and self-reported chemical intolerance (CSS score). Sensitivity and criterion were entered as predictor variables in the model and CSS score as the outcome variable. Age and sex were included in the model as control variables. All variables were entered simultaneously in a single block (forced entry). Before entering the variables in the model, standard assumptions were checked: influential cases, absence of multicollinearity, and homoscedasticity (Field, 2009; Menard, 1995). Influential cases were checked by use of the Cook’s distance test. The highest value for any participant was Cook’s D = .10, showing that there were no influential cases biasing the model (a value >1 likely indicates presence of a significant outlier; Field, 2009). Multicollinearity was checked by examining correlations between the predictor variables and by inspection of tolerance values. Multicollinearity may be present if the predictor variables are strongly correlated (*r ≥* .70) and/or if tolerance values are < .20 (Menard, 1995). The strongest correlation between any of the predictor variables was *r* = .352 (between the sensitivity variable and the criterion variable), and all tolerance values were > .20, indicating no issues with multicollinearity. Finally, homoscedasticity was assessed by plotting the standardized predicted scores against the standardized residual scores. The plots showed no clear patterns, indicating no heteroscedasticity in the data.

## Results

**Figure 2.**
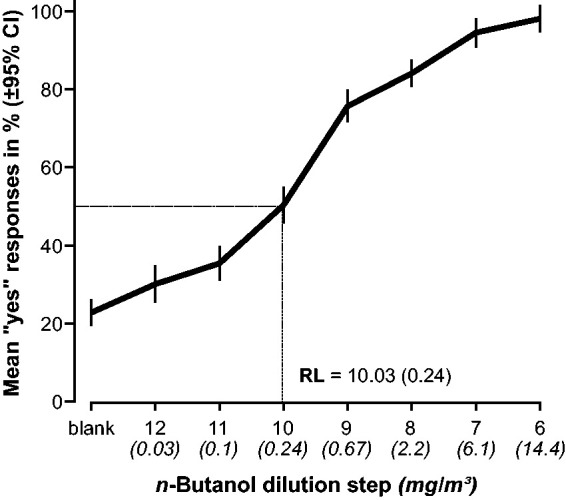
Group performance on the signal detection test. RL or stimulus threshold, that is, the dilution step (and mg/m^3^) representing 50% “yes” responses, here calculated for the entire sample. RL = Reiz Limen.

Distribution of responses for the different stimuli for the entire sample is presented in Figure 2. The Reiz Limen, or stimulus threshold (i.e., the concentration representing 50% “yes” responses), was slightly below dilution step 10 (10.03/0.24 mg/m^3^), with an SD of 1.29; a value close to previously reported thresholds assessed with dynamic olfactometry (Pacharra et al., 2020).

**Figure 3.**
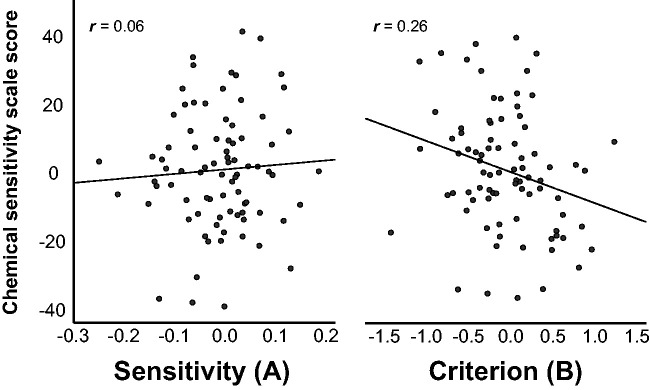
Partial plots from the regression model including sensitivity (A) and criterion (B) as predictors of chemical sensitivity scale score (sex and age entered as control variables).

Results from the regression analysis exploring sensitivity and criterion as predictors of chemical intolerance (CSS score) can be found in Table 1 and further illustrated as partial plots in Figure 3. As can be seen from the table, the criterion (B) significantly predicted CSS score, whereas sensitivity was not a significant predictor. Sex, but not age, was additionally a significant predictor of CSS.

**Table 1.** Multiple Regression With Age, Sex, Sensitivity, and Criterion as Predictor Variables and Chemical Sensitivity Scale (CSS) Score as Outcome.

Variable	β	*p*	*F*	*df*	*p*	*R* ^2^	Tolerance
CSS score			2.67	4, 77	.04	.12	
Age	–.033	.76					.96
Sex	.257	.02					.97
Sensitivity (A)	.063	.59					.87
Criterion (B)	–.266	.02					.86

## Discussion

The study revealed no association between self-reported chemical intolerance and sensory sensitivity. This is in line with previous results showing that chemical intolerance is not characterized by a particularly acute sense of smell (Caccappolo et al., 2000; Doty et al., 1988; Eis et al., 2008; Hummel et al., 1996; Kärnekull et al., 2011; Papo et al., 2006). Our results did, however, reveal that higher self-reported chemical intolerance is associated with a lower (more lenient) sensory criterion.

There are several possible explanations for the low criterion. For instance, it could reflect a local process such as an irritated or inflamed mucosa (Andersson, Nordin et al., 2009; Bascom et al., 1997; Claeson & Andersson, 2017; Meggs, 2017). If the mucosa cannot rinse away odorants from the receptors, this could mean that individuals with higher levels of chemical intolerance do in fact (correctly) experience stimuli when the odorant flasks has been removed, or when they contain blanks. Although outside the scope of the formal study procedure, it is in our experience not uncommon that participants in chemical exposure studies, especially those with chemical intolerance, spontaneously report that the odors linger for a long time after test sessions are over. The few studies that have assessed mucosal inflammation has not, in fact, found that chemical-intolerant individuals differ from controls in this regard (Dantoft, Skovbjerg, et al., 2015).

The results could also be due to an altered state of sensory nerves—that neurons are in a state of increased excitability or that they signal for a longer time than usual (Bascom et al., 1997; Bell et al., 1999; Meggs, 1999; Sullivan et al., 2001). Further, the criterion effect could be associated with altered adaptation patterns in sensory projection areas such as the piriform cortex, which is ordinarily characterized by rapidly decreasing activity after repeated or prolonged chemosensory exposure (Bensafi, 2011; Poellinger et al., 2001). These explanations should not be disregarded, yet they are also problematic to study. A problem in chemosensory research in general, and arguably chemical intolerance in particular, is that it is difficult to discern when a participant has returned to a proper sensory baseline. Increasing interstimulus intervals is one way to amend this problem but would in practical terms imply testing sessions of such durations that other aspects such as exhaustion would become troublesome factors.

Previous analyses of event-related potentials have shown that the early N1 peak does not habituate to the same degree in chemically intolerant when compared with controls (Andersson, Bende et al., 2009). As the N1 is assumed to reflect early sensory processing stages (Kok, 1997), this could be interpreted as an alteration in the signaling of chemosensory nerves. However, recent research has shown that habituation of the N1 and later peaks may be unrelated to early neural responses, for example, in the olfactory bulb, which do not generally express similar time-dependent signal decreases (Iravani et al., 2020). In addition, as previously illustrated by time plots of the piriform and orbitofrontal cortices following chemical exposure, the signal patterns of individuals with chemical intolerance do not seem to deviate from others, when assessed with functional magnetic resonance imaging (Andersson et al., 2014).

Evidence for significant alterations in early olfactory processing stages that could explain the observed lowered criterion is thus lacking but should not be discounted. Nevertheless, there are brain imaging results that are arguably more in line with the current findings, at processing stages beyond receptors, sensory nerves, and primary and secondary olfactory projection areas. Individuals who report that a reoccurring, invariant odor exposure is increasing in intensity over time, compared with those who report decreasing intensity, have higher self-rated chemical intolerance and express higher activity in the anterior insula and lower in the rostral anterior cingulate cortex (Andersson et al., 2017). These two nodes have been highlighted as important regulators of saliency, for example, for pain. As the criterion reflects a decision rule, we argue that the association between self-reported chemical intolerance and a low criterion could be an effect of increased saliency of chemosensory stimuli, which would relate the current results to altered connectivity in previously mentioned brain areas. These arguments should however be seen as hypotheses rather than conclusions.

It may be close at hand to interpret the altered criterion as a deviation from an optimal response. This is, however, not necessarily the case from an organismic perspective. Applying a low criterion implies a propensity of reacting when no stimulus is present but also a higher hit rate. Utilizing a lenient decision rule can thus be a sound strategy, for example, if the cost of misses is great. The criterion is often described as reflecting a judgment process that is under the conscious control of the observer (Wixted, 2019). Yet SDT measures can be applied to arbitrary systems, including the function of sensory receptors or other lower-tier networks. For the chemical intolerance debate, this has the implication that the current results do not solely support high-level explanatory theories.

Exposure studies on chemical intolerance are scarce, and outcomes are seldom clear. This is also the case in the current study. Even though we found a significant correlation between CSS and criterion, the effect size is relatively small (Cohen, 1992), which limits the conclusions that can be drawn. One reason for the weak effect in this and other studies is that the chemical intolerance concept itself is difficult to pin down. Criteria are problematically unspecific (McKeown-Eyssen et al., 2001), which makes for heterogeneous study samples. We therefore welcome further discussions regarding how to define chemical intolerance (Haanes et al., 2020), and especially endorse provocation-based criteria.

The study constitutes an important stepping-stone for further research into chemical intolerance. It would be close at hand to investigate the processes underlying the altered response criterion. For instance, it would be informative to investigate whether the results are specific to olfaction, or if it is an expression of a more lenient criterion also for other sensory stimuli. Further, future studies may also investigate whether chemical intolerance is associated with a more general inhibitory inability expressed in, for example, cognitive or affective domains. Using imaging methods may offer important insights into the neural bases of such possible commonalities. The results do not in themselves have practical implications for e.g., the treatment of chemical intolerance. However, we offer the possibly encouraging interpretation that the criterion results imply that chemical intolerance status is amenable to change to a greater degree than if the state/affliction was characterized by higher sensory sensitivity.

## Supplemental Material

sj-pdf-1-ipe-10.1177_2041669520978424 - Supplemental material for Chemical Intolerance Is Associated With Altered Response Bias, not Greater Sensory Sensitivity Click here for additional data file. Supplemental material, sj-pdf-1-ipe-10.1177_2041669520978424 for Chemical Intolerance Is Associated With Altered Response Bias, not Greater Sensory Sensitivity by Linus Andersson, Petra Sandberg, Elisabeth Åström, Moa Lillqvist and Anna-Sara Claeson in i-Perception
