# Supplementary material for: Chemical Intolerance Is Associated With Altered Response Bias, not Greater Sensory Sensitivity
Source: Iperception. 2020 Dec 20;11(6):2041669520978424. doi: 10.1177/2041669520978424 (PMC7754803; doi:10.1177/2041669520978424)
Supplement: sj-pdf-1-ipe-10.1177_2041669520978424 - Supplemental material for Chemical Intolerance Is Associated With Altered Response Bias, not Greater Sensory Sensitivity [file sj-pdf-1-ipe-10.1177_2041669520978424.pdf]

SUPPLEMENTARY INFORMATION for

Chemical intolerance is associated with altered response bias, not greater sensory sensitivity

Linus Andersson[1], Petra Sandberg[2], Elisabeth Åström[1], Moa Lillqvist[1], and Anna-Sara Claeson[1]

[1]Department of psychology, Umeå University

[2]Department of Radiation Sciences, Umeå University

Table S1

Self-reported health status of the sample

**Condition / diagnosis**	*n*
ADHD	0
Allergic rhinitis	9
Allergic asthma	8
Unexplained asthma	5
Atopic eczema	5
Back/joint/muscle pain	3
Burnout syndrome	3
Chronic fatigue	0
Chronic obstructive lung disease	0
Chronic sinusitis	1
Depression	6
Diabetes	2
Electrical hypersensitivity	0
Epilepsy	0
Fibromyalgia	6
Generalized anxiety	2
Heart problems	1
High blood pressure	7
Irritable bowel disorder	13
Multiple chemical sensitivity	0
Migraine	4
Multiple sclerosis	0
Noise sensitivity	1
Panic disorder	1
PTSD	1
Rheumatic disease	2
Sick building syndrome	1
Sensory hyperreactivity	2
